# A sensitive and scalable microsatellite instability assay to diagnose constitutional mismatch repair deficiency by sequencing of peripheral blood leukocytes

**DOI:** 10.1002/humu.23721

**Published:** 2019-03-06

**Authors:** Richard Gallon, Barbara Mühlegger, Sören‐Sebastian Wenzel, Harsh Sheth, Christine Hayes, Stefan Aretz, Karin Dahan, William Foulkes, Christian P. Kratz, Tim Ripperger, Amedeo A. Azizi, Hagit Baris Feldman, Anne‐Laure Chong, Ugur Demirsoy, Benoît Florkin, Thomas Imschweiler, Danuta Januszkiewicz‐Lewandowska, Stephan Lobitz, Michaela Nathrath, Hans‐Jürgen Pander, Vanesa Perez‐Alonso, Claudia Perne, Iman Ragab, Thorsten Rosenbaum, Daniel Rueda, Markus G. Seidel, Manon Suerink, Julia Taeubner, Stefanie‐Yvonne Zimmermann, Johannes Zschocke, Gillian M. Borthwick, John Burn, Michael S. Jackson, Mauro Santibanez‐Koref, Katharina Wimmer

**Affiliations:** ^1^ Institute of Genetic Medicine Newcastle University Newcastle upon Tyne United Kingdom; ^2^ Division of Human Genetics Medical University of Innsbruck Innsbruck Austria; ^3^ Institute of Human Genetics Biomedical Centre, University Hospital Bonn Bonn Germany; ^4^ Centre de génétique humaine Institut de pathologie et génétique (IPG) Gosselies Belgium; ^5^ Program in Cancer Genetics, Departments of Oncology and Human Genetics McGill University Montreal Quebec Canada; ^6^ Department of Human Genetics McGill University Montreal Quebec Canada; ^7^ Department of Medical Genetics McGill University Health Centre Montreal Quebec Canada; ^8^ Lady Davis Institute for Medical Research Jewish General Hospital Montreal Quebec Canada; ^9^ Department of Pediatric Hematology and Oncology Hannover Medical School Hannover Germany; ^10^ Department of Human Genetics Hannover Medical School Hannover Germany; ^11^ Department of Pediatrics and Adolescent Medicine Medical University of Vienna Vienna Austria; ^12^ The Genetics Institute, Rambam Health Care Campus, and The Ruth and Bruce Rappaport Faculty of Medicine, Technion – Israel Institute of Technology Haifa Israel; ^13^ Department of Pediatric Oncology Kocaeli University Kocaeli Turkey; ^14^ Department of Pediatrics CHR Citadelle Hospital, University of Liège Liège Belgium; ^15^ Pediatric Oncology, Helios‐Klinikum Krefeld Germany; ^16^ Department of Pediatric Oncology Hematology and Transplantation, Poznań University of Medical Sciences Poznań Poland; ^17^ Department of Pediatric Oncology/Pediatric Hematology Kliniken der Stadt Köln gGmbH, Children's Hospital Amsterdamer Strasse Köln Germany; ^18^ Pediatric Hematology and Oncology, Klinikum Kassel Kassel Germany; ^19^ Department of Pediatrics Pediatric Oncology Center , Technische Universität München Munich Germany; ^20^ Institut für Klinische Genetik, Olgahospital Stuttgart Germany; ^21^ Pediatrics Department University Hospital Doce de Octubre, i+12 Research Institute Madrid Spain; ^22^ Pediatrics Department Hematology‐Oncology Unit, Faculty of Medicine, Ain Shams University Cairo Egypt; ^23^ Department of Pediatrics Sana Kliniken Duisburg Duisburg Germany; ^24^ Hereditary Cancer Laboratory University Hospital Doce de Octubre, i+12 Research Institute Madrid Spain; ^25^ Research Unit Pediatric Hematology and Immunology, Division of Pediatric Hematology‐Oncology, Department of Pediatrics and Adolescent Medicine Medical University Graz Graz Austria; ^26^ Department of Clinical Genetics Leiden University Medical Center Leiden Netherlands; ^27^ Department of Pediatric Oncology Hematology and Clinical Immunology, University Children´s Hospital, Medical Faculty, Heinrich Heine University Duesseldorf Germany; ^28^ Department of Pediatric Hematology and Oncology Children's Hospital, University Hospital Frankfurt Germany

**Keywords:** Constitutional mismatch repair deficiency, genetic diagnostics, microsatellite instability, next‐generation sequencing, single molecule molecular inversion probes, variant classification

## Abstract

Constitutional mismatch repair deficiency (CMMRD) is caused by germline pathogenic variants in both alleles of a mismatch repair gene. Patients have an exceptionally high risk of numerous pediatric malignancies and benefit from surveillance and adjusted treatment. The diversity of its manifestation, and ambiguous genotyping results, particularly from *PMS2*, can complicate diagnosis and preclude timely patient management. Assessment of low‐level microsatellite instability in nonneoplastic tissues can detect CMMRD, but current techniques are laborious or of limited sensitivity. Here, we present a simple, scalable CMMRD diagnostic assay. It uses sequencing and molecular barcodes to detect low‐frequency microsatellite variants in peripheral blood leukocytes and classifies samples using variant frequencies. We tested 30 samples from 26 genetically‐confirmed CMMRD patients, and samples from 94 controls and 40 Lynch syndrome patients. All samples were correctly classified, except one from a CMMRD patient recovering from aplasia. However, additional samples from this same patient tested positive for CMMRD. The assay also confirmed CMMRD in six suspected patients. The assay is suitable for both rapid CMMRD diagnosis within clinical decision windows and scalable screening of at‐risk populations. Its deployment will improve patient care, and better define the prevalence and phenotype of this likely underreported cancer syndrome.

## 1 INTRODUCTION

Constitutional mismatch repair deficiency (CMMRD) is a highly penetrant cancer‐predisposition syndrome, caused by biallelic germline pathogenic variants affecting one of the four mismatch repair (MMR) genes: *MLH1*, *MSH2*, *MSH6*, or *PMS2*. CMMRD typically manifests in childhood or adolescence as one of a broad range of malignancies, primarily of the hematopoietic system and brain, as well as colorectal and other cancers associated with heterozygous germline MMR pathogenic variants (Lynch syndrome). Patients who survive their first malignancy have a high risk of metachronous disease (Wimmer et al., 2014). Current management guidelines recommend extensive surveillance from early childhood, with 1–2 yearly clinical examinations that include blood counts, optional abdominal ultrasound, brain MRI, and gastrointestinal endoscopy. These guidelines also advocate tailored treatment, such as extensive surgery to reduce the risk of metachronous disease (Durno et al., 2017; Tabori et al., 2017; Vasen et al., 2014), and there is evidence that immune checkpoint blockade therapy is effective in these patients (Bouffet et al., 2016). Aspirin intake may reduce cancer incidence in CMMRD, although bleeding risks must be considered (Leenders et al., 2018). Timely diagnosis of CMMRD is therefore important for appropriate patient management.

CMMRD also has a broad spectrum of benign and nonneoplastic features that can be shared with other tumor‐predisposition syndromes. Most prevalent among these are abnormal skin pigmentation reminiscent of neurofibromatosis type 1 (NF1) and Legius syndrome (Wimmer, Rosenbaum, & Messiaen, 2017). These features in childhood or adolescent cancer patients, as well as the type of malignancy, consanguineous parents, and a family history of Lynch syndrome cancers, are used for clinical diagnosis according to criteria developed by the Care for CMMRD (C4CMMRD) consortium (Wimmer et al., 2014). However, the phenotypic spectrum is broad and it is likely that the clinical manifestation of CMMRD is not fully characterized (Durno et al., 2017). Furthermore, the phenotypic overlap with NF1 and Legius syndrome has led to the acknowledgment of CMMRD as a legitimate, but presumably rare, differential diagnosis in children without malignancy who are suspected of these syndromes but lack the causative *NF1* or *SPRED1* variants (Suerink et al., 2019). Family history can also be misleading as pathogenic variants in *PMS2*, the gene implicated in more than 50% of CMMRD cases (Wimmer et al., 2014), have a much lower penetrance than other MMR variants in Lynch syndrome (Møller et al., 2017; Ten Broeke et al., 2018). Hence, the C4CMMRD criteria were designed to have high diagnostic sensitivity at the cost of specificity, and detection of pathogenic variants in both alleles of an MMR gene is required to confirm the diagnosis. Unfortunately, molecular genetic testing is not always definitive, and the diagnosis of CMMRD is frequently confounded by MMR variants of unknown significance (VUS) and pseudogenes of *PMS2* (De Vos, Hayward, Picton, Sheridan, & Bonthron, 2004), which is a recognized “dead zone” for diagnostic next‐generation sequencing (Mandelker et al., 2016).

The need to resolve diagnostic ambiguities has led to the development of highly sensitive microsatellite instability (MSI) assays, such as germline MSI (gMSI; Ingham et al., 2013) and ex vivo MSI (evMSI; Bodo et al., 2015), that detect low‐frequency microsatellite length variants in nonneoplastic tissues, a hallmark of CMMRD. gMSI is a simple PCR‐based assay using template DNA from peripheral blood leukocytes (PBLs), but analyses dinucleotide repeats that are insensitive to loss of MSH6 activity (Ingham et al., 2013). evMSI uses mononucleotide repeats that are sensitive for deficiency of any MMR protein, but requires a long‐term culture of primary lymphoblastoid cell lines and parallel analysis of alkylation tolerance (Bodo et al., 2015). There is a need for an MSI assay that is both accurate and simple, to assess the functionality of the MMR system within clinical decision windows. Furthermore, perhaps as a result of diagnostic difficulties, CMMRD is likely to be underdiagnosed. Recent epidemiological studies estimate that carriers of MMR pathogenic variants are relatively common (up to 1 in 279 of the general population), and that carriers of *PMS2* variants are the most common among these (Win et al., 2017). In addition, germline pathogenic variants in DNA repair genes, including those of the MMR system, are the most prevalent germline genetic cause of a variety of pediatric cancers (Gröbner et al., 2018). Despite this, only approximately 200 cases of CMMRD are known. Therefore, functional assays for CMMRD should, ideally, be applicable to patient screening at scale, to address its underdiagnosis.

We have previously described a novel panel of short microsatellites for accurate detection of MSI in colorectal cancers (CRCs), using high‐throughput sequencing and automated analysis (Redford et al., 2018). Here, we show that a subset of these markers, analyzed using molecular barcoding of sequencing reads to facilitate reduction of PCR and sequencing error, can detect low‐frequency microsatellite length variants in PBLs for the diagnosis of CMMRD.

## 2 PATIENTS AND METHODS

Thirty DNA samples extracted from PBLs were available from 26 genetically or functionally‐confirmed CMMRD patients (with three patients having multiple samples analyzed), and six samples were obtained from six suspected CMMRD patients with MMR missense VUS that lacked functional data to support pathogenicity (Table S1). This cohort constitutes approximately 15% of known cases (Wimmer et al., 2017) and includes biallelic pathogenic variants in each of the four MMR genes. Non‐CMMRD control PBL DNAs consented for use in assay development, were provided by 94 anonymized patients consulted for noncancer‐related conditions at the Medical University of Innsbruck. All CMMRD and control samples were collected and analyzed following ethical review by the Medical University of Innsbruck review board. Forty DNAs extracted from the PBLs of adult Lynch syndrome patients were received from the CaPP3 clinical trial (ISRCTN16261285) biobank and analyzed following an ethical review by the Newcastle University research ethics committee (REC reference 13/LO/1514). Full cohort details, including patient genotype, pertinent clinical history, and assay results, can be found in Table S2.

A panel of 24 short (7–12 bp), monomorphic, mononucleotide repeats (Table S3) were selected from the markers described by Redford et al. (2018). These were amplified from 100 ng of each sample using a multiplex of single molecule molecular inversion probes (smMIPs), following the protocol of Hiatt, Pritchard, Salipante, O'Roak, and Shendure (2013) with minor modifications: Herculase II Polymerase (Agilent, Santa Clara, CA) was used during extension and amplification steps, and amplification thermocycling used 98°C for 2 min, 30 cycles of 98°C for 15 s, 60°C for 30 s, and 72°C for 30 s, followed by 72°C for 2 min. Amplicons were purified using Agencourt AMPure XP Beads (Beckman Coulter, Brea, CA), pooled, and sequenced to a mean depth (±SD) of 3,642 ± 1,659 reads/marker/sample on a MiSeq (Illumina, San Diego, CA). Fastq files are available from the European Nucleotide Archive, accession number: PRJEB28798. Reads were aligned to reference genome hg19 using BWA v0.6.2 (Li & Durbin, 2010). smMIPs add molecular barcodes to amplicons to reduce sequencing error (Hiatt et al., 2013), and these were used to facilitate the detection of low‐frequency microsatellite length variants (Supporting Information S1).

The scarcity of CMMRD samples precluded classifier training and validation in independent cohorts, as described by Redford et al. (2018). As an alternative, we modeled the distribution of the relative frequency of reads containing the WT length of microsatellite (WT reads) for each marker in the first 40 control samples analyzed (see Results). To classify samples, we used these distributions to estimate the probability of an observed frequency of WT reads in a sample being greater than or equal to that of the control set. For each sample, the probabilities from the 24 markers were combined using Fisher's method to estimate the overall probability that a sample is from the control population. For ease of interpretation and presentation, we multiplied the decadic logarithm of this probability by minus one, and designated the transformed value as the score. Higher scores indicate increased MSI and, therefore, an increased likelihood of CMMRD. Details of the method are given in Supporting Information S2. The analysis was performed in R v3.3.1. The Beta distribution was used to model the control distribution of WT read frequencies, with distribution parameters calculated by the eBeta function of the ExtDist package. The metap package sumlog function was used to combine probabilities derived from these distributions by Fisher's method. R scripts are available upon request.

Transcript analysis was used to support variant pathogenicity for a subset of MMR missense VUS, following protocols described by Etzler et al. (2008).

## 3 RESULTS

**Figure 1:**
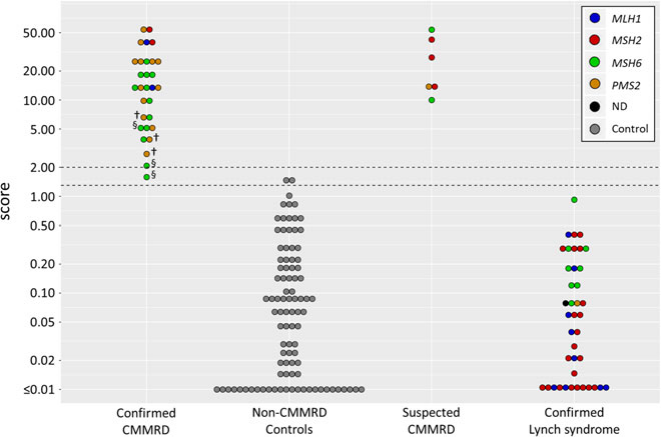
Score distribution of constitutional mismatch repair deficiency (CMMRD) and control samples. DNA samples from peripheral blood leukocytes of genetically‐confirmed CMMRD, non‐CMMRD control, suspected CMMRD, and Lynch syndrome patients were sequenced and scored (see Patients and methods). Suspected CMMRD patients had a clinical diagnosis and missense VUS detected in the indicated MMR gene (Table S1). Score thresholds at 1.30 and 2.00 are equal to 5% and 1% probability a sample is from a control population, respectively (horizontal dotted lines). The key indicates controls and the affected MMR gene in the CMMRD patients and Lynch syndrome patients (ND = affected MMR gene not disclosed). § Patient 8, blood sample collected during recovery from aplasia. † patients homozygous for hypomorphic *PMS2* pathogenic variant. MMR: mismatch repair; VUS: variants of unknown significance

An initial cohort of 40 controls, together with five CMMRD samples, were analyzed as proof of principle of the method (Supporting Information S2). Subsequently, a second cohort of the remaining 27 CMMRD patients and 54 controls were analyzed blind to sample status. Results from both cohorts are presented together in Figure 1. All samples from the 26 genetically or functionally‐confirmed CMMRD patients (score = 1.59–54.55) were separable from controls (score = 0.00–1.47; Figure 1). For CMMRD diagnosis, an a priori threshold of 5% probability that the sample is from the control population (score threshold = 1.30) achieved 100% sensitivity and 98% specificity across all samples (Figure 1). The more conservative threshold of 1% probability (score threshold = 2.00) failed to detect only one CMMRD sample (97% sensitivity, 100% specificity, Figure 1). However, this sample is one of three collected from Patient 8 when they were recovering from aplasia due to chemotherapy for T cell lymphoma (Figure 1, marked §). The other two samples also had low scores, but correctly identified patient 8 as CMMRD by the score threshold of 2.00. Patients 29, 30, and 31 are homozygous for a hypomorphic *PMS2* variant, shown to cause an attenuated CMMRD phenotype in the Nunavik Inuit population (Li et al., 2015). Their samples were correctly classified but had relatively low scores (2.76–5.90; Figure 1, marked †).

The six patients with MMR missense VUS suspected of CMMRD had scores ranging from 10.02 to 53.72 (Figure 1), consistent with their clinical diagnosis. For the two *MSH6* VUS, p.Asp439Gly and p.Tyr994Asn, and the *PMS2* VUS p.Gln700Arg, transcript analysis (Etzler et al., 2008) was used to exclude the presence of a different causative variant, such as deep intronic variants that lead to altered messenger RNA splicing, or regulatory variants that lead to the loss of expression of one allele that would be undetected by analysis of genomic DNA. The reclassification of these MMR missense VUS as (likely) pathogenic, at least in the context of CMMRD, should be considered (Table S1). Exclusion of a different causative variant by transcript analysis also supported the pathogenicity of the *MLH1* variant p.Val716Met in Patient 5, who has previously been confirmed as CMMRD (unpublished data) by the gMSI assay (Ingham et al., 2013). This variant, which has previously been identified as potentially disease‐causing in the context of CMMRD (Marcos, Borrego, Urioste, García‐Vallés, & Antiñolo, 2006), is classified as benign (Class 1) by the InSiGHT variant interpretation committee in the context of Lynch syndrome (Table S1).

As an independent confirmation of our results, we analyzed all CMMRD and control samples with the gMSI assay (Ingham et al., 2013). gMSI results were concordant with our findings, except for the 15 samples from patients with loss of MSH6 (Table S2), for which gMSI is known to be insensitive. Increased MSI has also been detected in the PBLs of Lynch syndrome patients relative to the general population using small pool PCR (Coolbaugh‐Murphy et al., 2010). To assess whether or not the assay would be able to discriminate between Lynch syndrome and CMMRD, we tested DNA extracted from the PBLs of 40 adult patients with confirmed pathogenic variants in one allele of an MMR gene. These patients scored 0.00–0.92, and were, therefore, distinct from the CMMRD patients analyzed and indistinguishable from controls (Figure 1).

## 4 DISCUSSION

We have previously shown that short (7–12 bp) mononucleotide repeats facilitate highly accurate MSI testing of CRC (Redford et al., 2018), and here show that they can detect low‐frequency microsatellite length variants in PBLs to diagnose CMMRD, using a smMIP‐based assay. The assay produces an easy‐to‐read score, equivalent to the probability that a sample is distinct from the non‐CMMRD, control population. Using an a priori score threshold of 2.00, only one CMMRD sample was missed, which was collected from a patient recovering from aplasia. Repeat samples from this patient were correctly classified as CMMRD but also had low scores, suggesting a reduced frequency of microsatellite length variants in their PBLs. This is consistent with the observation that MMR deficient hematopoietic stem cells with a higher burden of microsatellite mutations are associated with defective repopulation (Reese, Liu, & Gerson, 2003). An alternative explanation is that repopulating leukocytes have acquired fewer microsatellite length variants due to fewer cycles of DNA replication, following the polymerase slippage model of microsatellite mutation (Fan & Chu, 2007). It may, therefore, be appropriate to treat low scores in patients suspected of CMMRD who are aplastic, or recovering from aplasia, as inconclusive. Apart from this patient, we did not observe any effect of therapy on assay score: Samples from patients who were undergoing chemotherapy at the time of blood draw, or had previously had chemotherapy (Supp. Table S2), gave neither systematically higher or lower scores than other patients. This argues against the recent suggestion that the use of such agents may mask the mutational signature of MMR deficiency (Shuen et al., 2019).

The variety of genetically‐confirmed CMMRD patients included in the cohort allowed a limited analysis of variables that may affect score. Patients homozygous for a hypomorphic variant in *PMS2* had low scores, which may be a consequence of their residual MMR activity (Li et al., 2015). This suggests assay score may have prognostic value by indicating the penetrance of germline variants. An association between age and frequency of microsatellite length variants in PBLs has been detected in the general population (Coolbaugh‐Murphy, Xu, Ramagli, Brown, & Siciliano, 2005), and could lead to lower scores in younger CMMRD patients. We found no correlation between age and score in the CMMRD patients overall (*r* = 0.03, *p* = 0.89), although in patients from the same family, and therefore sharing the same MMR gene variants, we generally observed lower scores in younger compared with elder siblings (Table S2). For example, Patient 22 (score = 3.54 and 7.39), who had not presented with cancer, and was only 13 and 15 months old when blood samples were taken, was 8 years younger than their higher scoring sibling Patient 21 (score = 17.61). Variables such as clinical history and age may also contribute to the variation observed in the control scores. However, further analysis is beyond the scope of this study.

The smMIP protocol has a low per sample cost and is scalable (Hiatt et al., 2013), making our assay suitable for short turnaround diagnostics. Furthermore, and in contrast to assays of MMR function of patient cell extracts (Bodo et al., 2015; Shuen et al., 2019), our MSI assay could be used for high‐throughput screening of large patient cohorts or retrospective analysis of archived samples. The “missing” CMMRD cases may be identified by screening unselected pediatric cancer patients (Gröbner et al., 2018), and children suspected of NF1 or Legius syndrome who lack the causative *NF1* or *SPRED1* variants (Suerink et al., 2019). The assay also offers a means to investigate Lynch syndrome where CMMRD is a plausible explanation for an exceptional phenotype, given that it can distinguish between patients with mono‐ versus biallelic MMR variants. For example, it is recognized that pathogenic *PMS2* variants are less penetrant than other MMR gene variants in the context of Lynch syndrome (Møller et al., 2017; Ten Broeke et al., 2018), yet approximately 8% of CRCs in carriers of pathogenic *PMS2* variants are diagnosed before the age of 30 and in the distal colon, much earlier than the mean onset at 48 years in probands and distinct from the proximal location that is typical of Lynch syndrome (Goodenberger et al., 2016). Similarly, CMMRD patients with hypomorphic *PMS2* variants have a predominance of colorectal (i.e. not brain or hematological) malignancies that are frequently diagnosed in early adulthood (Li et al., 2015). Given the difficult diagnostic sequencing of *PMS2* (Mandelker et al., 2016), it is possible that some early onset Lynch syndrome cases supposedly caused by one pathogenic *PMS2* variant are actually CMMRD with an unrecognized hypomorphic allele.

Functional assays can clarify CMMRD diagnosis when MMR VUS are detected, but additional evidence is needed to confirm pathogenicity of the variant. For this study, we enhanced VUS classification by combining our assay with transcript analysis of the entire coding region of the relevant gene (Etzler et al., 2008), which excludes the presence of a different causative variant that may be missed by sequencing of genomic DNA. Through this approach, we confirmed that cancer predisposition is associated with the *MLH1* variant p.Val716Met despite its nonpathogenic classification in the context of Lynch syndrome. This shows the importance of tailoring MMR VUS classification depending on whether the associated disorder is autosomal dominant Lynch syndrome or autosomal recessive CMMRD.

In conclusion, our data confirm the results of Bodo et al. (2015), and Ingham et al. (2013) that assessment of MSI is an adequate measure of MMR function in nonneoplastic tissues for the diagnosis of CMMRD. In addition, our smMIP and sequencing‐based assay overcome the limitations of the previous MSI assays, providing a cheap and accurate test for CMMRD irrespective of which MMR gene is affected within clinical decision windows. It can also resolve ambiguous genetic testing results, and, combined with transcript analysis, can classify VUS in the context of CMMRD. Due to its low cost and scalability, the assay is also suited to high‐throughput screening of at‐risk populations. Hence, screening large patient cohorts with the presented assay and its systematic application in clinical practice would better describe the prevalence and phenotypic spectrum of CMMRD, as well as guide clinical management, genetic counseling, and germline genetic testing of patients and their families.

## CONFLICT OF INTERESTS

JB, MSJ, and MSK are named as inventors on the patent held by Newcastle University covering the markers used in this assay (Patent ID: PCT/GB2017/052488). JB receives an annual salary from QuantuMDx Ltd as their Chairman. JB, his spouse, and his son are shareholders of QuantuMDx Ltd. All other authors have no conflict of interests.

## Supporting information

Supporting Information

Supporting Information

Supporting Information

Supporting Information
