# Supplementary material for: A sensitive and scalable microsatellite instability assay to diagnose constitutional mismatch repair deficiency by sequencing of peripheral blood leukocytes
Source: Hum Mutat. 2019 Mar 6;40(5):649–55. doi: 10.1002/humu.23721 (PMC6519362; doi:10.1002/humu.23721)
Supplement: Supplementary file 1 — Supporting Information [file HUMU-40-649-s001.pdf]

Supp. Information S1: Reducing Sequencing Error using Molecular Barcodes

All reads originating from a single template DNA molecule within a smMIP amplification reaction will share the same molecular barcode. By grouping reads sharing the same molecular barcode, PCR and sequencing error can be corrected by assuming that errors will occur only in a minority of reads in a group, whereas true variants will be present in the majority or all reads in the group (Casbon *et al*, 2011; PMID: 21490082). Here, we were interested in the length of microsatellite detected. A single molecule sequence (smSequence) was defined as the microsatellite sequence that represents the majority of reads in a molecular barcode group. Molecular barcode groups were discarded if:

1. There were <2 reads within the group; with only one read in a group PCR and sequencing errors cannot be recognised.
2. There was no single microsatellite length that represented the majority (>50%) of reads in the group: without a majority for one length it is uncertain if variants in microsatellite length are due to PCR and sequencing error or a true variant.

A more stringent criterion of ≥3 reads within a group could have been used, however, this would have reduced the number of smSequences for analysis (Figure S1A).

We aimed to use smSequences to reduce the noise in detection of variants in microsatellite length so that low frequency, true variants would be detectable. Variants in microsatellite length from 40 control samples (Sample ID: 1-40) were considered errors of PCR or sequencing, based on the assumption that control template DNA from MMR proficient PBLs should not contain these variants. Using marker GM07 in a control sample (Sample ID: 40) as an illustrative example, the relative frequency of variants detected was reduced from 7.1% (98/1387) in all reads to 0.9% (2/230) in smSequences, which is equivalent to an 8.1-fold reduction in error by utilising molecular barcodes (Figure S1B).

A

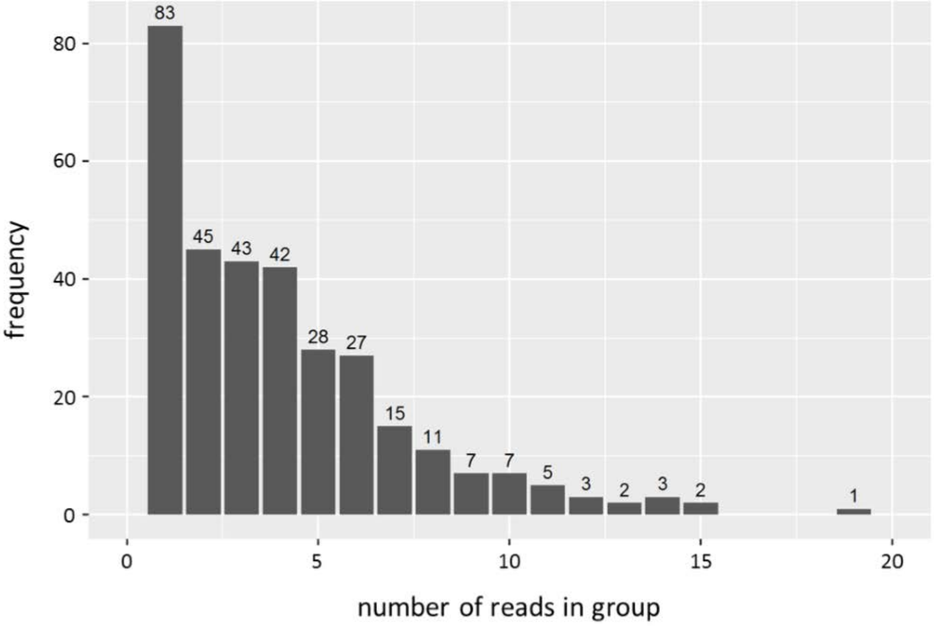

Figure S1A: The frequency distribution of molecular barcode groups by the number of reads within each group. Sequencing reads from microsatellite marker GM07 in a control sample (Sample ID: 40) were grouped according to molecular barcode. Each group was classed by the number of reads within it, and the frequency of groups containing different numbers of reads was determined.

B

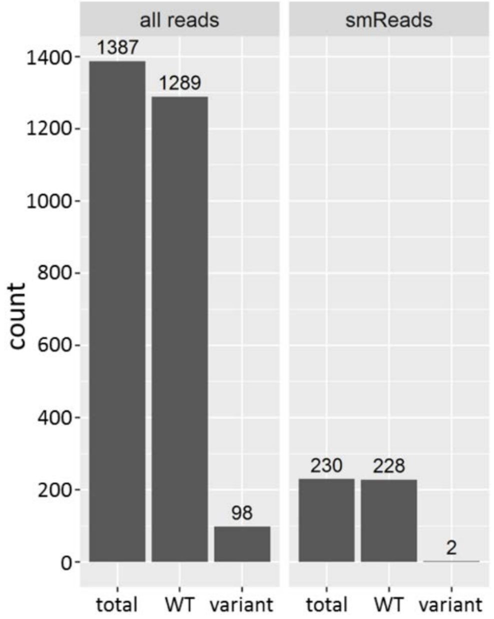

Figure S1B: Count of different microsatellite lengths, using either all reads irrespective of molecular barcode, or single molecule sequences (smSequences). Sequencing reads from microsatellite marker GM07 in a control sample (Sample ID: 40) were counted, using either "all reads" irrespective of molecular barcode, or reads grouped by molecular barcode and summarised in one smSequence. Counts of total, wild type (WT), and variant microsatellite lengths are shown.

To test whether use of molecular barcodes significantly reduced error in microsatellite sequencing, we analysed the relative frequency of variants in microsatellite length in 40 control samples (Sample IDs: 1-40) in all 24 microsatellite markers, using either all reads or smSequences (Figure S1C). To prevent variants common to all PBLs of a sample, such as those inherited through the germline or acquired in early cell divisions, from affecting the analysis of error rate, observations where the relative frequency of length variants was >0.4 (equivalent to prWT <0.6, see Supp. Information S2) were excluded; across the 960 observations from these 40 controls (i.e. 24 microsatellite markers in 40 samples) only 6 of these variants were detected and excluded. By using the relative frequency of variants in microsatellite length in controls as a quantitative measure of error rate, again assuming that PBLs from controls contain no variants in microsatellites, it was evident that different markers had different error rates (Figure S1C, top panel). We then looked at the change in error rate when smSequences were used rather than all reads, using the equation:

$$fold.change = \frac{error\ rate\ of\ smSequences}{error\ rate\ of\ all\ reads}$$

Fold-change above 1 means smSequences have increased error rate of microsatellite length detection, fold-change below 1 means smSequences have decreased error rate of microsatellite length detection.

All markers showed a reduction in error rate in all samples when smSequences were used except for GM09 in four samples, with the vast majority showing a two-fold or greater reduction in error (fold-change in error rate < 0.5, Figure S1C, middle panel). In some observations, use of smSequences removed all error in microsatellite length detection such that all smSequences contained a wild type (WT) microsatellite length, giving an infinite-fold reduction in error (fold-change in error rate = 0, Figure S1C, middle panel); this was more frequent in less error prone markers (Figure S1C, compare middle and top panels). Excluding those samples with a fold-change = 0, the magnitude of fold-change is correlated with the error rate of the marker ($r_s = -0.29$, $p < 10^{-10}$), showing that smSequences facilitate a greater reduction of errors in more error-prone markers. For 15/24 markers, the majority of the control samples analysed had a fold-change that was significant ($p < 0.05$) by Fisher's exact test (Figure S1C, bottom panel). We concluded that smSequences would improve detection of true, low frequency variants in microsatellite length, and therefore would improve detection of low-level MSI associated with CMMRD.

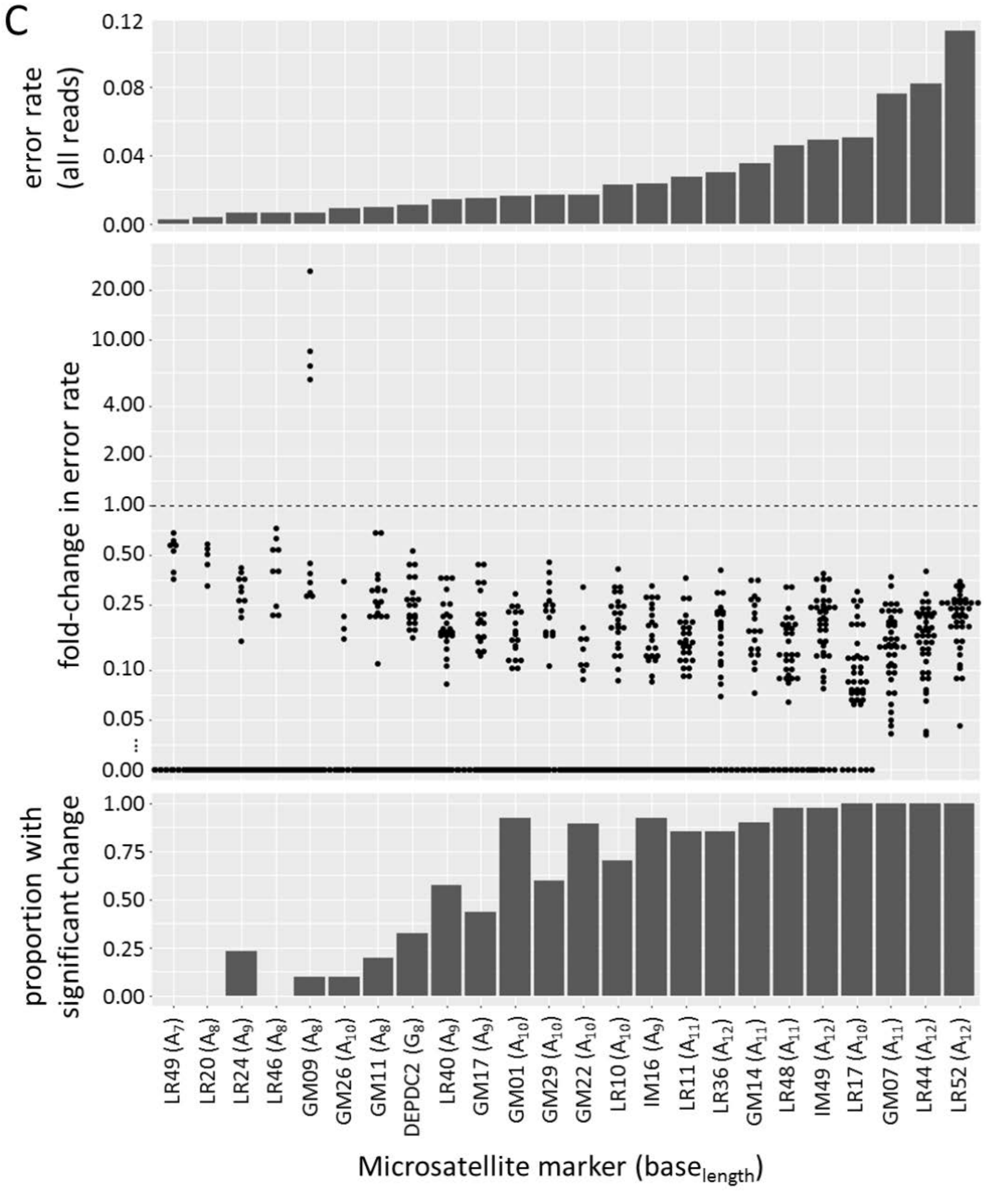

Figure S1C: Using single molecule sequences (smSequences) reduces the error in detection of variants in microsatellite length. Top panel: Microsatellites are listed from left to right in order of increasing error rate, as measured by the relative frequency of microsatellite length variants from all reads, averaged (mean) across the 40 control samples analysed (Sample IDs: 1-40). **Middle panel:** Fold change in error rate observed when smSequences were analysed rather than all reads irrespective of molecular barcode (fold-change <1 represents a reduction in error rate). **Bottom panel:** The proportion of fold-changes in error rate that are statistically significant for each marker using Fisher’s exact test.

Supp. Information S2: Analysis Method

A simple and automatable method was developed that would quantify deviation of a sample from a control distribution. To establish this method, 40 control samples (Sample IDs: 1-40) were amplified and sequenced by the MSI assay. 5 CMMRD samples (Sample IDs: A-E) were also sequenced, to be used as proof of principle for the method. Reads with the same molecular barcode were grouped, based on the assumption that they originate from the same template molecule of DNA. A single molecule sequence (smSequence), containing the microsatellite length present in the majority of reads, was defined for each group to reduce sequencing error (Supp. Information S1). For each sample and microsatellite marker, read data was represented in a single value: the relative frequency of smSequences that contained a wild type length of microsatellite (prWT). Out of 960 markers analysed (24 markers x 40 controls), 954 had a prWT frequency >0.83. However, 6 had a prWT <0.6, suggesting they were heterozygous for rare germline microsatellite length variants.

For each marker, the prWT in the 40 controls was modelled by a Beta distribution, excluding any samples containing suspected inherited length variants (prWT < 0.6) so that the analysis would only account for somatic microsatellite mutation. Using the fitted distributions, the probability of an observed prWT being equal to or greater than the prWT from a control population was determined. For each sample, the probabilities from the 24 markers were combined into one probability, excluding germline length variants (prWT < 0.6), using Fisher's method. The combined probability from Fisher's method was then converted into an easy to read score for each sample using the following equation:

$$\text{score} = -\log_{10}(\text{combined probability})$$

Examples of observed prWT, probabilities, and sample score from a control and CMMRD patient can be found in Table S2A. Higher scores can be interpreted as an increased frequency of length variants across the 24 microsatellite markers relative to the control population, and therefore an increased likelihood of CMMRD. As score is a probability, *a priori* score thresholds can be set. In this study, we selected 95% and 99% probabilities as two thresholds to consider, equivalent to scores of 1.30 and 2.00, respectively.

	Sample ID: 16 (control)		Sample ID: C (CMMRD)	
**Marker**	**prWT**	**probability**	**prWT**	**probability**
**DEPDC2**	1.000	1.000	0.994	0.055
**GM01**	0.997	0.155	0.997	0.168
**GM07**	0.992	0.711	0.936	0.000
**GM09**	0.996	0.205	0.999	0.259
**GM11**	0.997	0.166	0.997	0.143
**GM14**	1.000	1.000	0.966	0.001
**GM17**	1.000	1.000	0.997	0.160
**GM22**	0.997	0.044	0.998	0.122
**GM26**	1.000	1.000	0.989	0.000
**GM29**	1.000	1.000	0.991	0.025
**IM16**	0.997	0.286	0.989	0.014
**IM49**	0.986	0.182	0.973	0.008
**LR10**	0.996	0.276	0.981	0.002
**LR11**	0.996	0.302	0.987	0.015
**LR17**	0.995	0.458	0.966	0.000
**LR20**	1.000	1.000	0.997	0.027
**LR24**	0.529	NA	0.996	0.030
**LR36**	1.000	1.000	0.989	0.036
**LR40**	0.997	0.189	0.990	0.003
**LR44**	0.989	0.590	0.951	0.001
**LR46**	0.996	0.048	0.995	0.030
**LR48**	0.989	0.100	0.977	0.007
**LR49**	1.000	1.000	1.000	1.000
**LR52**	0.962	0.086	0.897	0.000
		**combined p: 0.32**		**combined p: 4.6×10^{-28}**
		**score: 0.49**		**score: 27.34**

Table S2A: Conversion of observed proportion of smSequences containing a wild type microsatellite length (prWT) to a probability and sample score. Observed prWT can be converted into a probability that it is equal to or greater than expected of a control population using, for each marker, the Beta distribution of prWT in controls. prWT and probability are shown for each marker in one control and one CMMRD sample. Note the exclusion of a germline length variant in sample 16, marker LR24 (prWT < 0.6). One combined probability is calculated from the 24 markers using Fisher's method, and is converted into a score for each sample using $score = -\log_{10}(combined\ probability)$.
